# Optimized management of urolithiasis by coloured stent-stone contrast using dual-energy computed tomography (DECT)

**DOI:** 10.1186/s12894-019-0459-3

**Published:** 2019-04-30

**Authors:** Giuseppe Magistro, Patrick Bregenhorn, Bernhard Krauß, Dominik Nörenberg, Melvin D’Anastasi, Anno Graser, Philipp Weinhold, Frank Strittmatter, Christian G. Stief, Michael Staehler

**Affiliations:** 1 Department of Urology, Ludwig-Maximilians-University of Munich, Marchioninistrasse 15, 81377 Munich, Germany; 2 Department of Radiology, Ludwig-Maximilians-University of Munich, Munich, Germany; 3 Siemens Healthcare GmbH, Research and Development, Forchheim, Germany; 4 Gemeinschaftspraxis Radiologie München, Munich, Germany

**Keywords:** Urolithiasis, Stone disease, Dual-energy computed tomography, Stent-stone-contrast

## Abstract

**Background:**

We analysed in vitro the appearance of commonly used ureteral stents with dual-energy computed tomography (DECT) and we used these characteristics to optimize the differentiation between stents and adjacent stone.

**Methods:**

We analysed in vitro a selection of 36 different stents from 7 manufacturers. They were placed in a self-build phantom model and measured using the SOMATOM® Force Dual Source CT-Scanner (Siemens, Forchheim, Germany). Each sample was scanned at various tube potentials of 80 and 150 peak kilovoltage (kVp), 90 and 150 kVp and 100 and 150 kVp. The syngo Post-Processing Suite software program (Siemens, Forchheim, Germany) was used for differentiation based on a 3–material decomposition algorithm (UA, calcium, urine) according to our standard stone protocol.

**Results:**

Stents composed of polyurethane appeared blue and silicon-based stents were red on the image. The determined appearances were constant for various peak kilovoltage (kVp) values. The coloured stent-stone-contrast displayed on DECT improves monitoring, especially of small calculi adjacent to indwelling ureteral stents.

**Conclusion:**

Both urinary calculi and ureteral stents can be accurately differentiated by a distinct appearance on DECT. For the management of urolithiasis patients can be monitored more easily and accurately using DECT if the stent shows a different colour than the adjacent stone.

## Background

Urolithiasis is a common bothersome condition with a prevalence of 4–20% and an upward trend is reported in developed countries [1, 2]. Non-contrast enhanced computed tomography (NCCT) is the standard for diagnosing patients with acute flank pain [3]. Low-dose NCCT has emerged as the imaging technique of first choice in the acute setting. It provides both excellent sensitivity of 97% and specificity of 95% for the detection of urinary calculi [4]. NCCT determines accurately location, size, density and skin-to-stone distance, all of which are relevant determinants for treatment decision. With the introduction of technical innovations like dual-energy computed tomography (DECT) acquisition of additional information on chemical stone composition is now possible. The attenuation difference produced by two different x-ray energy spectra is utilized to differentiate uric-acid (UA) calculi from non-uric-acid (non-UA) stones. The post-processing software applied for analysis uses a 3-material decomposition algorithm, which characterizes a calculus as a mixture of UA, calcium and urine. Based on this algorithm, material-specific chromatic image-pixels with an attenuation ratio similar to UA are coloured red, those similar to non-UA stones appear blue. This classification is achieved with high accuracy, which is supported by a reported sensitivity of up to 100% [5–12]. The fact can be decisive for optimal management as in case of UA stones pharmacological chemolitholysis is preferred to interventional approaches [3].

Of note, ureteral stents are also assigned a specific colour according to their material composition on DECT. The imaging of small stones adjacent to a ureteral stent is a common pitfall in the current diagnostic assessment. The option to display stent and stone in contrasting colour may help to optimize the detection. The colour-coded characteristics of calculi based on chemical composition are well documented, whereas for ureteral stents they do not. The determinants underlying the phenomenon of red and blue stents on DECT scans have not been elucidated yet. In the current work it was our main objective to characterize in vitro the appearance of different stents from various manufacturers using DECT.

## Methods

### In vitro setting

**Fig. 1:**
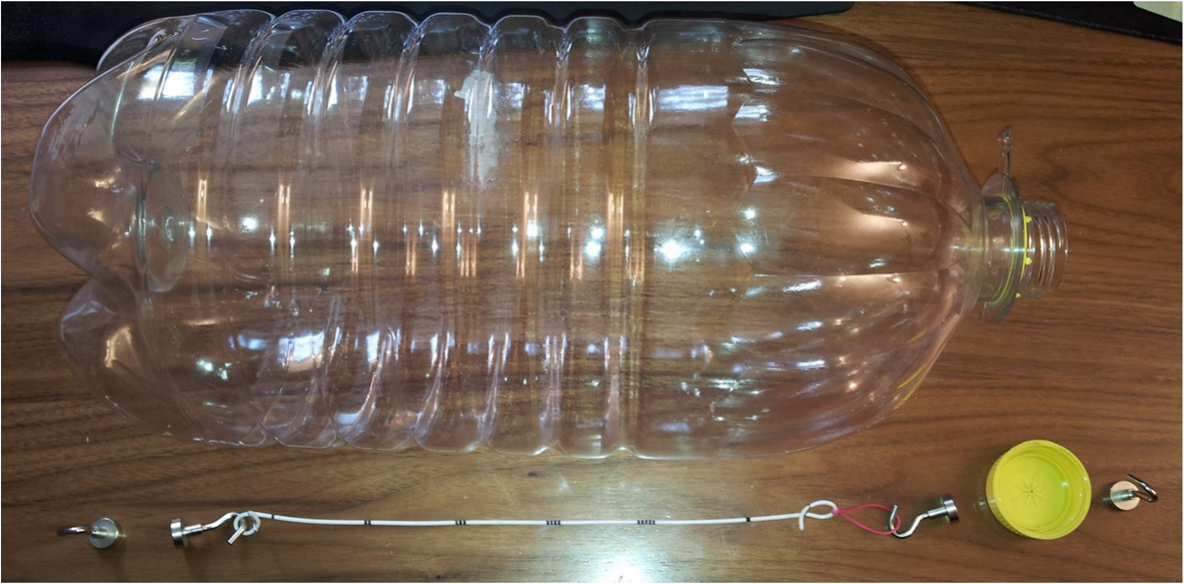
A container was filled with water at body temperature (36 °C). Stents were fixed with clips at both ends and spanned throughout the phantom model for measurements

We purchased 36 commonly used stents from 7 manufacturers and analysed them on the third generation SOMATOM® Force Dual Source CT-Scanner (Siemens, Forchheim, Germany). We used a phantom model measuring 45 cm × 20 cm × 20 cm (height x width x depth) and filled it with water at body temperature (36 °C) (Fig. 1). The model itself and experimental settings had no influence on CT performance and analysis. Stents were fixed with clips, spanned throughout the phantom model and consecutive measurements were performed. Each sample was scanned at various tube potentials of 80 and 150 peak kilovoltage (kVp), 90 and 150 kVp and 100 and 150 kVp. We affixed a calculus of 2 mm in diameter of known chemical composition (calcium oxalate monohydrate; blue on DECT scan) to blue and red stents and repeated measurements.

### CT protocol and post processing

The images were acquired on a SOMATOM® FORCE Siemens computed tomography (CT) scanner (Siemens, Forchheim, Germany) in dual-energy mode. The patients underwent imaging with 100/Sn150 kVp reflecting our current institutional dual-energy protocol. Continuous non-contrast images were obtained from the level of the diaphragm to the pubic symphysis. A slice thickness of 4 mm and interval of 4 mm were chosen to be consistent with our clinical practice. The images were reconstructed on a multimodality WorkPlace (Siemens) using CT syngo Post-Processing Suite software, version VA 50A (Siemens). Reconstructions used a 0.75-mm slice thickness and 0.7-mm interval, with a QR40 convolution kernel for optimal data analysis. DECT allows differentiating between uric acid (UA) and non-UA stones, which are colour-coded based on a post-processing algorithm. The syngo Post-Processing Suite software program utilizes a 3-material (UA, calcium, and urine) decomposition algorithm to assign colour (red or blue) based on the ratio of X-ray attenuations from the two tube potentials. Materials that more closely resemble the dual-energy characteristics (DEC) of UA stones are depicted in red and those that more closely resemble the DEC of non-UA stones are depicted in blue.

## Results

**Fig. 2:**
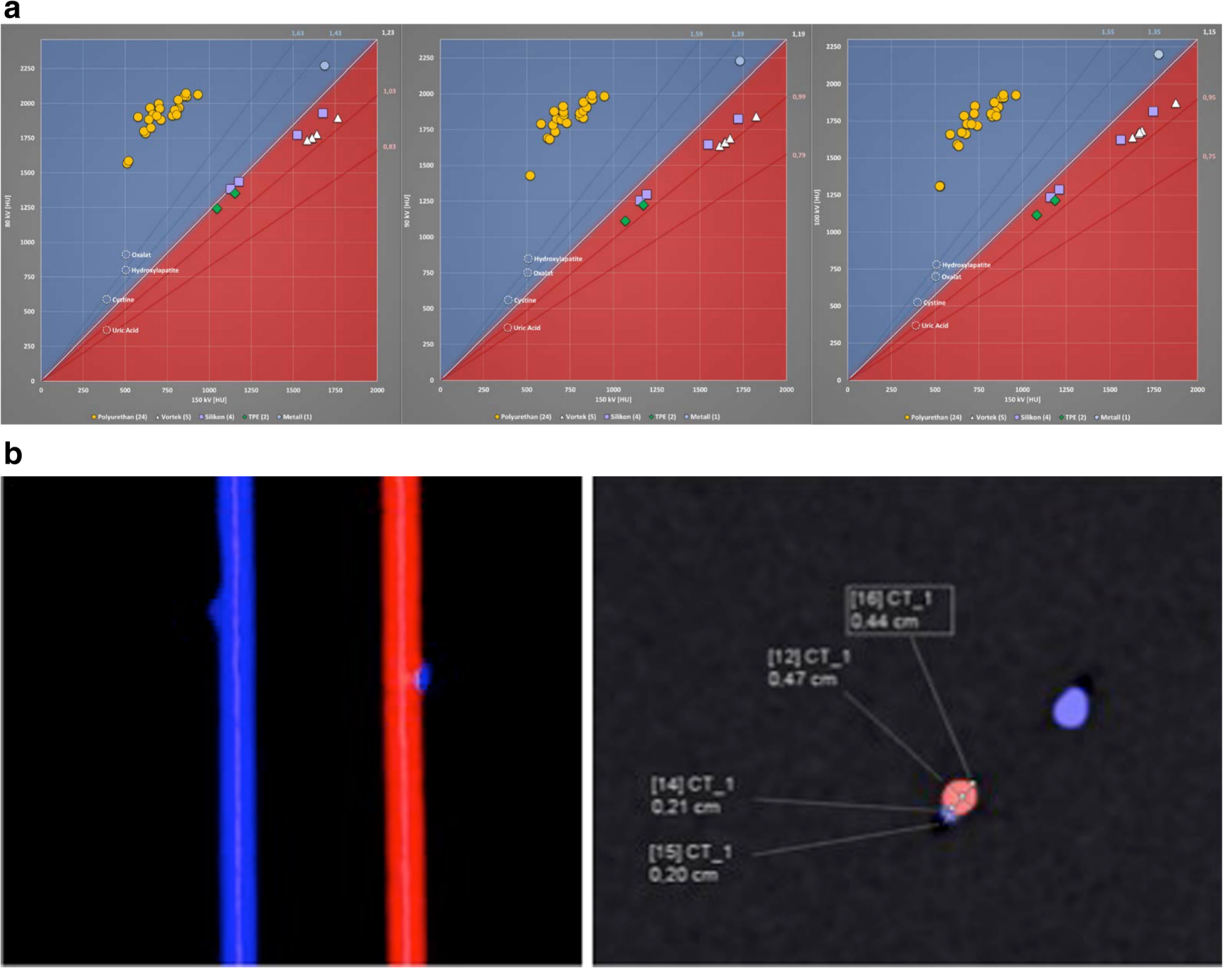
**a**. Results acquired for various tube potentials (80/150 kVp; 90/150 kVp; 100/150 kVp). Respective Hounsfield units (HU) were measured and plotted. Stents above the cut-off of 1,15 appeared blue and samples below the cut-off appeared red. Reference values for known stone types are depicted as well. Polyurethane-based samples were blue, silicone-based stents were red. **b.** A pure calcium oxalate monohydrate stone of 2 mm (blue) was affixed to a blue and red stent on DECT. The enhanced stent-stone-contrast obtained for the red stent allows clear differentiation of the stone from the adjacent stent. For the blue stent a contour irregularity of the stent is indicative of stone presence, better displayed on coronal than sagittal images

Results are demonstrated in Fig. 2a. All polyurethane-based stents showed a blue appearance on DECT, whereas stents composed of silicone were red. The assigned colours were constant over various kVp values. The determined colour according to material composition of various stents from the same manufacturer was consistent throughout our measurements (Table 1). As shown in Fig. 2b, placing a pure calcium oxalate monohydrate stone (blue) of 2 mm in size next to a ureteral stent of blue colour makes it difficult to discern the fragment on both coronal and sagittal reformatted DECT images. Now, using a silicone-based stent, which appears red on DECT, the presence of the stone could be easily detected due to a clear stent-stone-contrast.

**Table 1:** A random selection of 36 various stents was purchased from 7 manufacturers. Material composition and reference numbers for each model are displayed. Stents are classified according to their appearance as red or blue on DECT

Manufacturer	RED	BLUE
Uromed		Hydropur (polyurethane, 4640–28) Hydropur (polyurethane, 4687–28) Heparius (polyurethane, 4377–28) Heparius (polyurethane, 4387–28)
Urotech		Yellow-Star (polyurethane, TU-360628) Yellow-Star (polyurethane, EP-360628) Green-Star (polyurethane, EG-480628) White-Star (polyurethane, ES-370628) White-Star (polyurethane, ES-570730)
Cook	C-Flex Towers (TPE, 037732) Universa Soft (TPE, USH-722-T1) Black Silicone (silicone, 133,624)	Universa UFH (polyurethane, UFH-772-T1) Resonance (metal, RMS-060022-R)
Rüsch		Superglide DD (polyurethane, 334,841) Superglide integral (polyurethane, 334,248) Integral Stent Set (polyurethane, 334,201) DD-Ureterstent (polyurethane, 334,801)
Optimed		Optisoft (polyurethane, 3004–2400) Optipur (polyurethane, 3034–2400) Optisplint (polyurethane, 3064–2400) Carbosoft (polyurethane, 3090–2400)
Coloplast/Porges	Vortek Tumor Stent (Vortek, BCCG75) Vortek (Vortek, ACB576) Vortek Hydrogel (Vortek, BCFA75) Vortek Hydrogel (Vortek, BNFA75) Vortek Mono-J (Vortek, ACA207) Silicone (silicone, AJ4275) Silicone (silicone, AJ4A75) Silikon Pyelostent (silicone, AJ4Y85) Silikon Stenostent (silicone, AJ4W85)	PU-R (polyurethane, AC4D75) PU-R (polyurethane, AC4B75) PU-S (polyurethane, AC4274) Biosoft Duo (polyurethane, BNAA75)
IMP		Tumorstent (polyurethane, S137996070300)

## Discussion

Information on chemical stone composition optimizes management of urolithiasis in many ways. As mentioned above, DECT assures identification of UA stones with high accuracy, thus efficient chemolitholysis should be preferred rather than interventional treatment, if it is clinically appropriate and safe. However, the overall incidence of UA stones was estimated to be 11.7% for men and 7% for women [13]. Thus, the clinical impact on management is actually rather low. Furthermore, the unique ability to determine stone types may prevent inefficient treatment options. Extracorporeal shockwave lithotripsy (ESWL) achieves good results for renal stones ≤20 mm, but shockwave-resistant stones composed of calcium oxalate monohydrate or cystine are known negative predictors for success [14]. Both stone types can be identified with high diagnostic accuracy using DECT [15]. In these cases multiple ineffective procedures can be avoided in favour of a more effective treatment modality such as percutaneous nephrolithotomy (PNL).

A distinct appearance of ureteral stents on DECT has been observed, but a systematic analysis of this phenomenon is lacking [16]. In the current study we investigated in vitro a random selection of 36 various samples from 7 manufacturers and we were able to match appearance (blue or red) to material composition of a stent. In our setting polyurethane-based stents were always blue on DECT, stents composed of silicone appeared always red. We further demonstrated that an enhanced stent-stone-contrast by selecting a stent in a different colour than the adjacent stone facilitates safe and easy detection, even for 2 mm calculi. This enhanced stent-stone-contrast allows clear differentiation in both coronal and sagittal images. Without colour coding, as in NCCT, only a contour irregularity may be suggestive of an adjacent stone, which becomes speculative when calculi decrease in size. This diagnostic obstacle may be overcome using color-coding based on a post-processing algorithm as applied in DECT. We demonstrated that a pure calcium oxalate monohydrate stone (blue) of 2 mm could be clearly differentiated from a silicon-based stent (red). Next to a blue stent, the presence of the stone was indicative by a poorly defined irregularity, which was more prominent on the coronal scan. The analysis of other 2 mm stones with different composition but a distinct colour on DECT scans, could always be detected accurately when the stent appeared in contrasting colour.

This feature may be of value in various clinical scenarios for the management of urolithiasis. The advantage of DECT technology aids urologists in stent selection according to stone appearance and assures accurate monitoring of stone patients. It has to be acknowledged, that stenting is indicated only in case of obstructive pyelonephritis, anuria and analgesia not achieved medically. The impact of our findings still needs to be evaluated for the clinical management.

The early results of DECT imaging for the management of urolithiasis are promising, however, due to the infancy of this technology relevant issues still need to be addressed. Certain pitfalls have been reported including reduced specificity for small calculi < 3 mm and patients with large body habitus [17]. With the development of third-generation scanners and modification of protocol settings and post-processing the capacity and potential of the technology is still advancing [18].

Certain limitations of this study need to be acknowledged. First of all, although we present the analysis of the largest stent collection to date with convincing consistency, we cannot exclude that stents from different manufacturers may display a diverse phenotype. Certain coatings might have a profound impact on appearance on DECT. Second, we present mainly in vitro data. Prospective, clinical trails are necessary to confirm our first results in stone patients and to evaluate the true benefit for management.

## Conclusions

Polyurethane-based stents are blue and stents composed of silicone appear red using DECT imaging. The enhanced stent-stone-contrast using DECT imaging is an additional feature that may be helpful for stent selection. Whenever available, it can contribute to an easy and accurate monitoring of stone patients. The coloured stent-stone-contrast displayed on DECT improves detection, especially of small calculi < 3 mm next to indwelling ureteral stents. Wherever DECT is available, urologists are encouraged to screen for blue and red stents in their institution and to choose ureteral stents according to stone appearance.

## Abbreviations

DECT: Dual-energy computed tomography
kVp: peak kilovoltage
NCCT: Non-contrast enhanced computed tomography
PNL: Percutaneous nephrolithotomy
UA: Uric acid
